# Succession of Bacterial Communities in a Seasonally Stratified Lake with an Anoxic and Sulfidic Hypolimnion

**DOI:** 10.3389/fmicb.2017.02511

**Published:** 2017-12-14

**Authors:** Muhe Diao, Ruben Sinnige, Karsten Kalbitz, Jef Huisman, Gerard Muyzer

**Affiliations:** ^1^Department of Freshwater and Marine Ecology, Institute for Biodiversity and Ecosystem Dynamics, University of Amsterdam, Amsterdam, Netherlands; ^2^Department of Ecosystem and Landscape Dynamics, Institute for Biodiversity and Ecosystem Dynamics, University of Amsterdam, Amsterdam, Netherlands

**Keywords:** anoxia, freshwater bacteria, ecological succession, 16S Amplicon sequencing, co-occurrence analysis, stratified lake

## Abstract

Although bacteria play key roles in aquatic food webs and biogeochemical cycles, information on the seasonal succession of bacterial communities in lakes is still far from complete. Here, we report results of an integrative study on the successional trajectories of bacterial communities in a seasonally stratified lake with an anoxic hypolimnion. The bacterial community composition of epilimnion, metalimnion, and hypolimnion diverged during summer stratification and converged when the lake was mixed. In contrast, bacterial communities in the sediment remained relatively stable over the year. Phototrophic *Cyanobacteria* and heterotrophic *Actinobacteria, Alphaproteobacteria* and *Planktomycetes* were abundant in the aerobic epilimnion, *Gammaproteobacteria* (mainly *Chromatiaceae*) dominated in the metalimnion, and *Chlorobi, Betaproteobacteria, Deltaproteobacteria*, and *Firmicutes* were abundant in the anoxic sulfidic hypolimnion. Anoxic but nonsulfidic conditions expanded to the surface layer during fall turnover, when the epilimnion, metalimnion and upper hypolimnion mixed. During this period, phototrophic sulfur bacteria (*Chromatiaceae* and *Chlorobi*) disappeared, *Polynucleobacter* (*Betaproteobacteria*) and *Methylobacter* (*Gammaproteobacteria*) spread out from the former meta- and hypolimnion to the surface layer, and *Epsilonproteobacteria* dominated in the bottom water layer. *Cyanobacteria* and *Planktomycetes* regained dominance in early spring, after the oxygen concentration was restored by winter mixing. In total, these results show large spatio-temporal changes in bacterial community composition, especially during transitions from oxic to anoxic and from sulfidic to nonsulfidic conditions.

## Introduction

Freshwater lakes provide vital ecosystem services to human society. As key players in biogeochemical cycles and water quality, bacteria in freshwater lakes have been studied extensively (Eiler and Bertilsson, 2004; Kent et al., 2007; Nelson, 2009; Shade et al., 2012). Many lakes in the temperate zone are stratified during the summer period, with a warmer upper layer called the epilimnion, and a colder, darker and sometimes anaerobic deeper layer known as the hypolimnion. Global warming will extend the range and duration of seasonal stratification in many lakes, which is likely to affect the abundances, species composition, and seasonal succession of bacteria in these different water layers (Huisman et al., 2004; Paerl and Huisman, 2008; North et al., 2014; Visser et al., 2016).

As a central theme in ecology, community succession has attracted numerous studies (Clements, 1916; Walker and Moral, 2003). One classic view, advocated by Clements (1916), is that succession largely proceeds as a deterministic orderly process and therefore successional trajectories should be highly predictable. An alternative view, developed by Gleason (1926), is that succession is based on the independent responses of a large number of individual organisms. Therefore, Gleason attributed a much greater role to chance events, and argued that succession is much less predictable than advocated by the Clementsian view. Both viewpoints have been extensively debated over the years, particularly in studies of succession of plants (Inouye and Tilman, 1995; Kreyling et al., 2011) and animals (Chase, 2010). Seasonal succession of abundant bacterial taxa in lakes, such as *Cyanobacteria* and *Betaproteobacteria*, has been investigated using fluorescence *in situ* hybridization (FISH) and whole-community fingerprinting (Eiler and Bertilsson, 2004; Kent et al., 2007; Salcher et al., 2008; Nelson, 2009; Šimek et al., 2014). In recent years, next generation sequencing of DNA provided enhanced taxonomic resolution and hence further insight into community succession of bacteria in the epilimnion of seasonally stratified lakes (e.g., Eiler et al., 2012; Okazaki and Nakano, 2016). Comparative studies of bacterial succession in different water layers are still relatively rare (e.g., Shade et al., 2008; Garcia et al., 2013; Yu et al., 2014; Okazaki and Nakano, 2016), but could shed more light on the impact of seasonal stratification on successional trajectories.

In this study, we present a comprehensive investigation of the trajectories of bacterial succession in different water layers and the sediment of Lake Vechten in The Netherlands. Lake Vechten is a eutrophic seepage lake that becomes stratified in an aerobic epilimnion and an anaerobic, sulfidic hypolimnion during summer and autumn, while it is well mixed during winter and early spring (Best et al., 1978; Blaauboer, 1982; Steenbergen and Verdouw, 1982). There are no streams or rivers connected to Lake Vechten, and hence bacterial succession in the lake is not affected by changes in source populations upstream. The seasonal stratification and relatively stable hydrological conditions therefore make Lake Vechten an excellent model system to investigate bacterial succession.

The main objectives of this study were: (i) to compare the composition and seasonal succession of bacterial communities in different layers of the water column and in the sediment, (ii) to identify environmental variables that affect bacterial succession, and (iii) to infer ecological relationships between community members and environmental variables. For this purpose, samples from different water layers and the sediment of Lake Vechten were collected monthly over one year, and 16S rRNA gene amplicon sequencing was employed to determine the composition and successional trajectories of the bacterial communities.

## Materials and Methods

### Study Site, Sampling, and General Analyses

**FIGURE 1:**
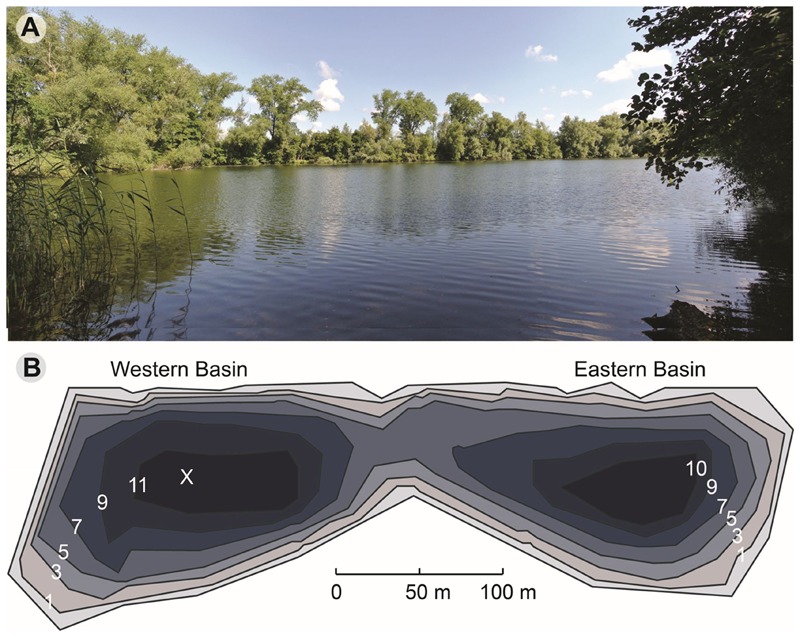
Lake Vechten. **(A)** Photo of the lake (taken on July 19, 2013). **(B)** Depth map of Lake Vechten and the sampling point (X). Updated from Steenbergen and Verdouw (1982).

Lake Vechten (52°04′N, 5°05′E) is located in the center of The Netherlands, a few km southeast of the city of Utrecht. It consists of two basins with a total surface area of 4.7 ha, and has a maximum depth of 11.9 m (**Figure 1**). Vertical profiles of temperature, dissolved oxygen (DO), chlorophyll *a*, photosynthetically active radiation (PAR), specific conductivity and pH of lake water were measured *in situ* using a multiprobe Hydrolab DataSonde 4a (Hydrolab Corporation, Austin, TX, United States). Water samples from every meter depth were collected monthly or biweekly from March 2013 to April 2014 from the Western basin. Water was pumped via a hose connected to the Hydrolab Datasonde, so that the water samples matched the conditions measured by the Hydrolab Datasonde at that particular depth. Water samples were filtered through 0.20 μm nylon membrane filters (Millipore, GNWP) to collect bacterial cells for DNA-based community analysis. Filters were frozen immediately and stored at –20°C, until further processing. Sediment samples (top 10 cm) were collected monthly with a box-corer from the same location.

Subsequent to previous filtration, dissolved organic carbon (DOC) was measured by total organic carbon analyzer (TOC-V_CPH_, Shimadzu, Japan), while ammonium (NH_4_^+^), nitrite (NO_2_^-^), nitrate (NO_3_^-^), total dissolved inorganic nitrogen (DIN), sulfate (SO_4_^2-^), phosphate (PO_4_^3-^), and chloride (Cl^-^) were measured by an auto-analyzer (SAN^++^, Skalar, The Netherlands). For sulfide (S^2-^) measurements, lake water was filtered through 0.20 μm polyethersulfone membrane filter and fixed by zinc acetate (10% w/v) immediately in the field. Afterward, sulfide was measured in the laboratory according to methylene blue spectroscopic method (Trüper and Schlegel, 1964). The data were visualized with Ocean Data View (version 4.6.5.; Schlitzer, 2002).

### DNA Extraction

DNA was extracted from the bacterial cells on the filters by using the PowerSoil DNA Isolation Kit according to manufacturer’s instructions (Mo Bio, Laboratories Inc., United States). The concentration of extracted DNA was quantified with the Qubit dsDNA BR Assay Kit (Invitrogen, United States).

### 16S rRNA Gene Amplicon Sequencing and OTU Assignments

We first profiled the PCR-amplified 16S rRNA genes of all 189 water samples and 11 sediment samples by denaturing gradient gel electrophoresis (DGGE). Based on the DGGE profiles and measured vertical stratification pattern of Lake Vechten, we selected 51 water samples and 4 sediment samples for 16S rRNA gene amplicon sequencing (Supplementary Table S1). These 55 samples covered the complete variation in microbial community composition detected by DGGE. Sequencing was performed on an Illumina MiSeq system by Research and Testing Laboratory (Lubbock, TX, United States). The primer pair S-D-Bact-0341-b-S-17 (5′-CCTACGGGNGGCWGCAG-3′) and S-D-Bact-0785-a-A-21 (5′-GACTACHVGGGTATCTAATCC-3′) were used to generate paired-end sequence reads, covering the V3–V4 region of the 16S rRNA gene (Herlemann et al., 2011).

Data analysis started with a denoising step in which short sequences, singletons, and noisy reads were removed, followed by a chimera check, in which chimeric sequences were removed. In order to determine the taxonomic information for each remaining sequence, the sequences were first quality checked and demultiplexed. Subsequently, the sequences were clustered into operational taxonomic units (OTUs) using the UPARSE algorithm program (Edgar, 2013). The centroid sequence from each cluster is then run against a database of high-quality sequences derived from the NCBI database using the USEARCH global alignment algorithm. The global search method uses a mixture of the USEARCH global search algorithm along with a python program to determine the actual taxonomic assignment that is assigned to each read. From the top 6 sequence matches a confidence value was assigned to each taxonomic level (phylum, class, order, family, genus and species). Once confidence values were assigned for each sequence an RDP formatted output file was generated for the final analysis in USEARCH. Subsequently, the data were entered in the diversity analysis program that takes the OTU/dereplication table output from sequence clustering along with the output generated during taxonomic identification and began the process of generating a new OTU table with the taxonomic information tied to each cluster.

The 16S rRNA gene amplicon sequences have been deposited as dataset SAMN06314865-SAMN06314918 in the Sequence Read Archive (SRA) of the National Center for Biotechnology Information (NCBI).

### Statistical Analysis

Non-metric multidimensional scaling (NMDS) analysis was used to ordinate data using the software program PAST (Hammer et al., 2001). NMDS analysis was based on Bray-Curtis similarities calculated between samples using the relative abundances of bacterial species. The ordination was plotted as a two-dimensional graph to enhance interpretability.

Environmental parameters, except for pH, were log (x+1)-transformed for redundancy analysis. The data were fitted to the redundancy analysis (RDA) model using the R software package (version 3.0.3). Environmental parameters were used as explanatory variables and bacterial taxa were the response variables in the RDA model. The explanatory variables were reduced by eliminating collinearity through calculation of the variance inflation factors (VIF) using the R function VIF in the ‘car’ package (Fox and Weisberg, 2011). Explanatory variables were analyzed step-wise until only those with a VIF < 10 remained. To further reduce the model to the most significant explanatory variables, we used the Ordistep function in the R package ‘vegan’ to apply forward selection permutation analysis and reveal those terms that contributed significantly to the model (Oksanen et al., 2013). Significance was determined using a permutation test with a multivariate pseudo-F statistical test and 9999 permutations (Zuur et al., 2009).

### Co-occurrence Network Analysis

Taxon-taxon and taxon-environment co-occurrence networks were constructed with the Cytoscape plug-in software program CoNet (Faust and Raes, 2012; Faust et al., 2012). An ensemble of correlation measures of bootstrap and renormalization approach, which can reduce false positive and compositionality biases, was employed to identify co-occurrence and mutual exclusion interactions. Correlation or dissimilarity scores were calculated using Spearman and Kullback–Leibler dissimilarity (Lima-Mendez et al., 2015). Potential false-positive correlations were further controlled by using the ReBoot procedure with 4000 permutations (Faust and Raes, 2012; Faust et al., 2012). The resulting distribution was run with 4000 bootstraps. Finally, a false discovery rate of 5% (*q* ≤ 0.05; Benjamini and Hochberg, 1995) was applied to the *P*-values of all correlations to control for multiple comparisons. In each analysis, the *P*-value for correlations was combined across Spearman and Kullback–Leibler dissimilarity measures.

## Results

### Seasonal Variation of Environmental Conditions

**FIGURE 2:**
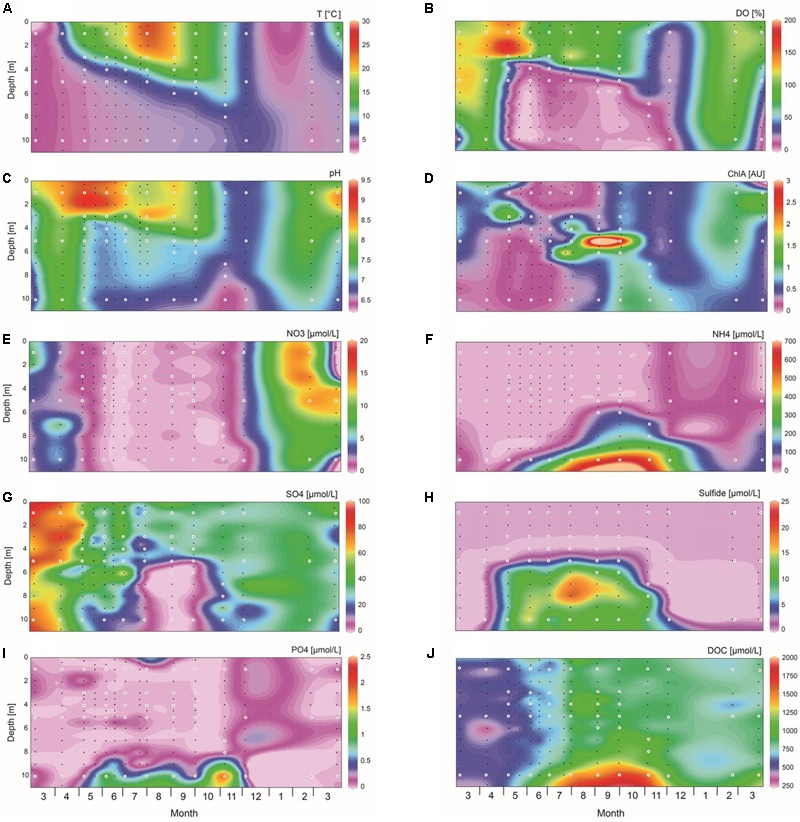
Spatio-temporal dynamics of environmental variables. **(A)** Temperature, **(B)** dissolved oxygen (DO), **(C)** pH, **(D)** chlorophyll *a*, **(E)** nitrate, **(F)** ammonium, **(G)** sulfate, **(H)** sulfide, **(I)** phosphate, **(J)** dissolved organic carbon (DOC). Environmental parameters were measured for 189 water samples indicated by black and white dots, while microbial community analysis was performed on 51 water samples indicated by the white dots. The graphs were created with Ocean Data View, version 4.6.5.

Vertical profiles of physical and chemical parameters showed distinct seasonal variation in Lake Vechten (**Figure 2**). Temperature was homogeneous over the entire water column in winter and early spring. From April onward temperature in the surface layer increased, creating a typical stratified lake consisting of an epilimnion, metalimnion and hypolimnion (**Figure 2A**). During the phytoplankton spring bloom in April and May the epilimnion was supersaturated with oxygen (**Figure 2B**). Subsequently, the epilimnion remained aerobic and maintained a relatively high pH of 8–9 during the summer stratification, while the hypolimnion became anaerobic and had a much lower pH of 6.5–7.2 from May onwards (**Figures 2B,C**). Chlorophyll *a* in the top layer (0–4 m depth) was high in April and May, decreased in June and stayed relatively low until the next spring (**Figure 2D**), while a high concentration of chlorophyll *a* developed in the metalimnion from July until October. Interestingly, the entire water column became low in dissolved oxygen and pH when the lake was mixed during the fall turnover in November and December (**Figures 2B,C**). At 10 m depth the pH remained lower and the ammonium concentration higher than at shallower depths, indicating that surface mixing by wind action and convective cooling did not fully extend to the deepest parts of the lake during fall turnover (**Figures 2C,F**). The oxygen concentration recovered to near saturation throughout the entire water column in March, and also the other physico-chemical parameters were essentially uniform over depth, indicating that mixing did reach the deeper parts of the lake in early spring.

The nitrate concentration was <1 μM across the whole water column during the stratification period, and increased to 15 μM in spring (**Figure 2E**). Nitrite was hardly detected in the water column (data not shown). The ammonium concentration remained low throughout the year in the top 5 m of the water column, but accumulated in the deeper part of the hypolimnion during the stratification period where it reached 635 μM in October (**Figure 2F**). Sulfate concentrations were highest (∼70 μM) in early spring, decreased to <10 μM in the hypolimnion from August to October, and increased again after the stratification period (**Figure 2G**). Sulfide was only detected in the hypolimnion from May to November, reaching concentrations of >15 μM in August and September (**Figure 2H**). Phosphate was at or below the detection limit for most of the time, except for a slightly higher concentration (1–2 μM) just above the lake sediment during the stratification period (**Figure 2I**). The DOC concentration was ∼500 μM from March to May, and then increased especially in the deeper part of the hypolimnion (**Figure 2J**).

### Community Composition

**FIGURE 3:**
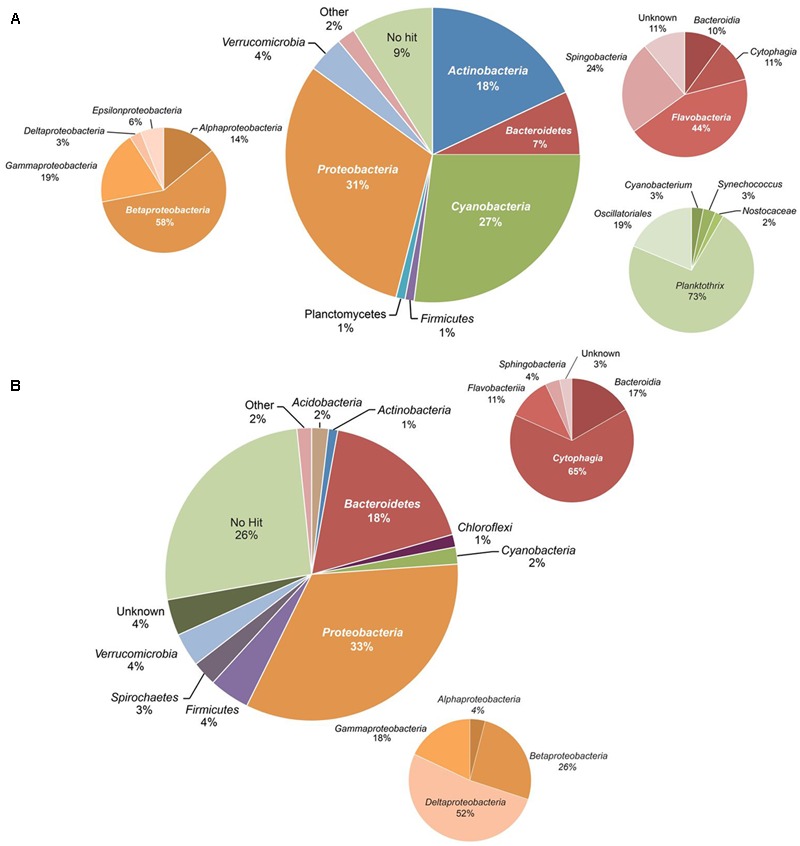
Overall composition of the bacterial community based on all 16S rRNA amplicon sequences. **(A)** Water column, **(B)** sediment. The smaller pie charts show the taxonomic composition within the *Proteobacteria, Bacteroidetes*, and *Cyanobacteria*.

High-quality sequences were received for 54 of the 55 samples selected for 16S rRNA gene amplicon sequencing, with a total of 4802 OTUs. The bacterial community composition showed major variation in space and time (Supplementary Figure S1). As a first step, we calculated average percentages of bacterial taxa in the water column and sediment. In the water samples, most OTUs belonged to the phyla *Proteobacteria* (31%), *Cyanobacteria* (27%), *Actinobacteria* (18%), *Bacteroidetes* (6.8%), *Verrucomicrobia* (4.0%), *Planctomycetes* (1.4%), and *Firmicutes* (1.3%) (**Figure 3A** and Supplementary Table S2). Within the *Proteobacteria, Betaproteobacteria* formed the most abundant class, followed by *Gammaproteobacteria, Alphaproteobacteria, Epsilonproteobacteria*, and *Deltaproteobacteria*. The main genera of *Cyanobacteria* in Lake Vechten were members of the family *Oscillatoriales*, and in particular the filamentous cyanobacterium *Planktothrix.* The phylum *Bacteroidetes* consisted of members of the classes *Flavobacteria, Sphingobacteria, Cytophagia* and *Bacteroidia*, while *Verrucomicrobia* were composed of the *Verrucomicrobiae* and *Opitutae*. Other bacterial phyla, such as *Acidobacteria, Chlorobi, Chloroflexi, Lentisphaerae, Spirochaetes*, and *Fusobacteria* were present at low average abundances (<0.5%). However, taxa with a low average abundance could be more abundant in some samples, for instance *Firmicutes* constituted up to almost 8% of the total bacterial community in some samples, while their average percentage was only 1.3% (Supplementary Table S2).

The composition of bacterial communities in the sediment was different from the water column (**Figure 3B**). *Proteobacteria* (33%) and *Bacteroidetes* (18%) were the most abundant phyla in the sediment. In particular, *Deltaproteobacteria* and *Cytophagia* formed the main classes of *Proteobacteria* and *Bacteroidetes*, respectively. Other phyla, such as the *Firmicutes, Spirochaetes, Verrucomicrobia, Cyanobacteria*, and *Actinobacteria*, were present at lower abundance (1–5%).

### Seasonal Succession of Bacterial Communities

**FIGURE 4:**
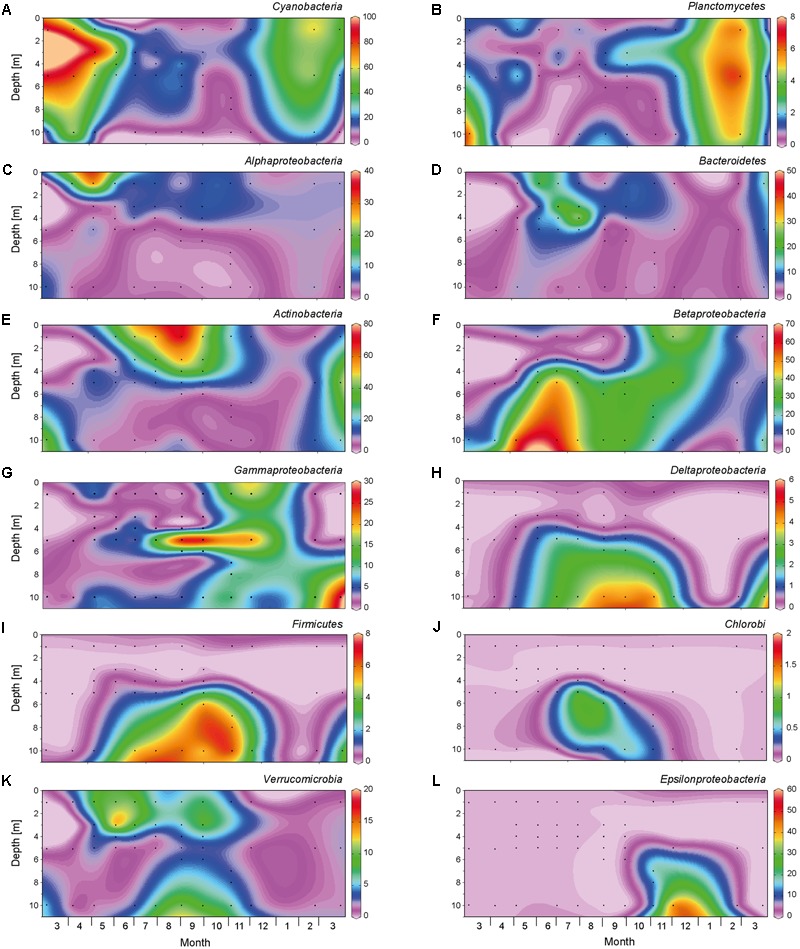
Spatio-temporal dynamics of major bacterial taxa based on 16S rRNA gene amplicon sequences. The graphs show relative abundance of **(A)** *Cyanobacteria*, **(B)** *Planctomycetes*, **(C)** *Alphaproteobacteria*, **(D)** *Bacteroidetes*, **(E)** *Actinobacteria*, **(F)** *Betaproteobacteria*, **(G)** *Gammaproteobacteria*, **(H)** *Deltaproteobacteria*, **(I)** *Firmicutes*, **(J)** *Chlorobi*, **(K)** *Verrucomicrobia*, **(L)** *Epsilonproteobacteria*. Black dots indicate the sampling points for the 16S rRNA gene sequences. The graphs were created with Ocean Data View, version 4.6.5.

**FIGURE 5:**
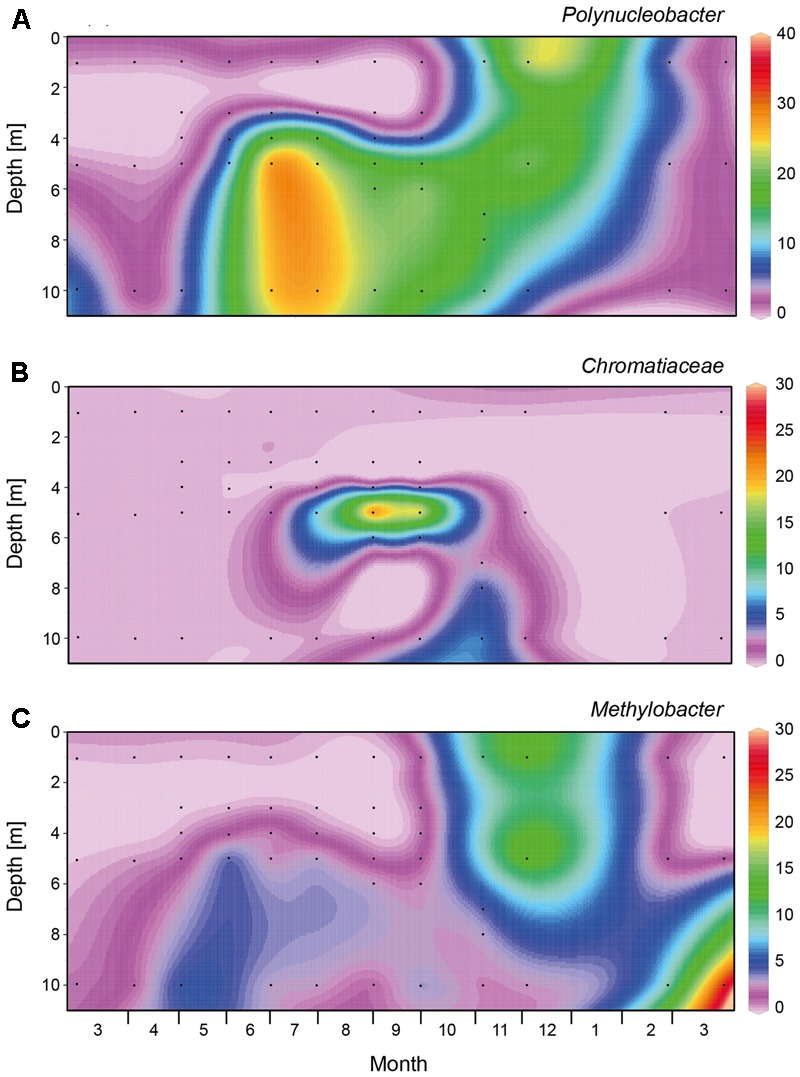
Spatio-temporal dynamics of abundant genera and family within the *Beta-* and *Gammaproteobacteria* based on 16S rRNA gene amplicon sequences. **(A)** *Polynucleobacter*, **(B)** *Chromatiaceae*, **(C)** *Methylobacter*. Black dots indicate the sampling points for the 16S rRNA gene sequences. The graphs were created with Ocean Data View, version 4.6.5.

From March to May 2013, a large bloom of *Cyanobacteria* (primarily *Planktothrix* spp.) dominated the bacteria (**Figure 4A** and Supplementary Figure S1). In the spring of 2014, *Planktothrix* bloomed again. Similarly, *Planctomycetes* reached highest abundances in early spring, before the onset of summer stratification (**Figure 4B**). At the onset of stratification, in May 2013, *Alphaproteobacteria* became abundant in the top meter of the water column (**Figure 4C**), followed by a high abundance of *Bacteroidetes* in June 2013, which were in turn replaced by *Actinobacteria* as the most dominant bacterial group (up to 80%) in the epilimnion during summer (**Figures 4D,E**). *Betaproteobacteria* (mainly *Polynucleobacter*) dominated in the meta- and hypolimnion during summer stratification (**Figures 4F, 5A**), while *Gammaproteobacteria* (mainly purple sulfur bacteria of the *Chromatiaceae*) reached high abundances in the metalimnion in September and October (**Figures 4G, 5B**). *Deltaproteobacteria, Firmicutes*, and *Chlorobi* (green sulfur bacteria) were present in the hypolimnion during summer stratification, but largely disappeared during fall turnover (**Figures 4H–J**). *Verrucomicrobia* were present in both the epilimnion and hypolimnion, but not in the metalimnion, during the stratification period (**Figure 4K**). *Polynucleobacter* (*Betaproteobacteria*) and *Methylobacter* (*Gamma proteobacteria*) spread out from the former metalimnion and hypolimnion into the surface layer during fall turnover in November and December (**Figures 5A,C**). *Epsilonproteobacteria* became abundant in the deeper water layers in November and December, but were rare (<1%) otherwise (**Figure 4L**).

The bacterial community composition in the sediment showed much less dramatic changes than in the water column. *Proteobacteria* (mainly *Deltaproteobacteria, Betaproteobacteria*, and *Gammaproteobacteria*) and *Bacteroidetes* (mainly *Cytophagia*) dominated the sediment community throughout the year (Supplementary Figure S1).

**FIGURE 6:**
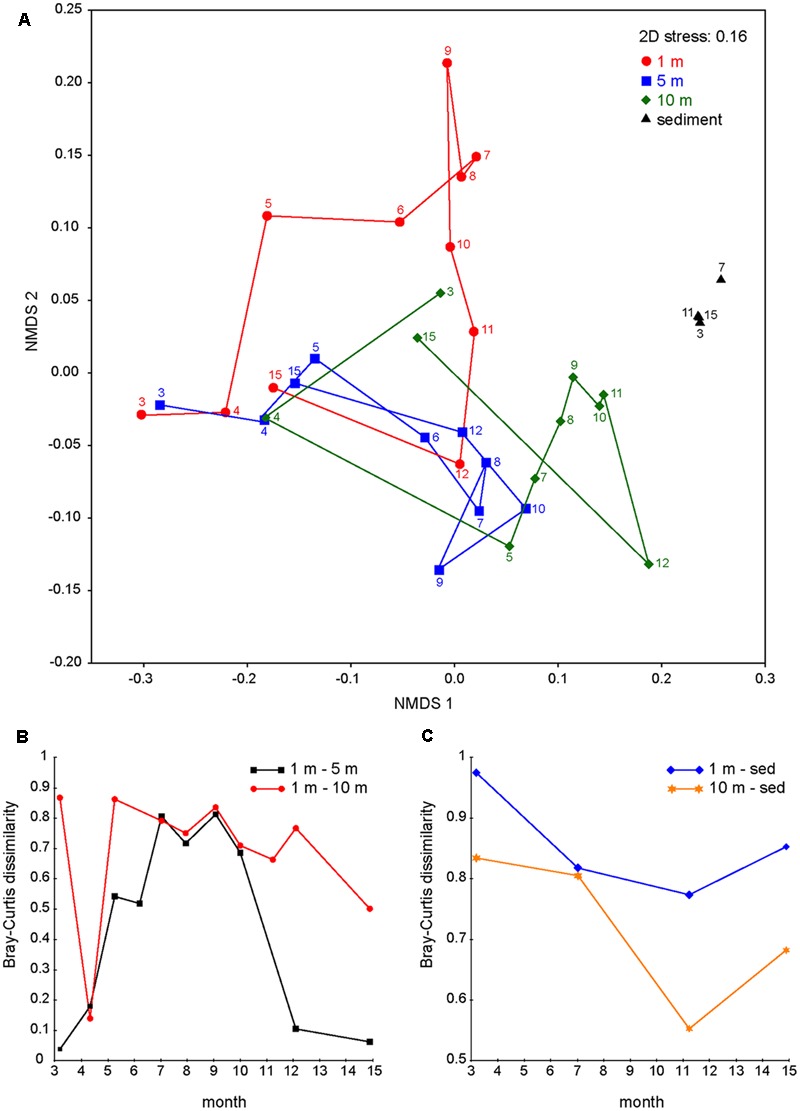
Divergence and convergence of bacterial communities in different water layers. **(A)** Non-metric multidimensional scaling (NMDS) plots of bacterial communities at 1, 5, 10 m depth and in the sediment throughout the year. Samples were grouped according to depth (1, 5, 10 m and sediment) and connected by time sequence. Numbers indicate months (e.g., 3 is March 2013 and 15 is March 2014). **(B)** Bray–Curtis dissimilarities between bacterial communities from different water layers (1 and 5 m; 1 and 10 m). **(C)** Bray–Curtis dissimilarities between bacterial communities from different water layers and the sediment (1 m and sediment, 10 m and sediment).

To visualize the succession of bacterial communities from different water layers, the OTUs of the epilimnion (1 m), metalimnion (5 m), hypolimnion (10 m), and sediment were classified at the species level and then used to compute a Bray–Curtis similarity matrix that was subsequently ordinated into two dimensions using NMDS (**Figure 6A**). The bacterial communities of the three water layers were very similar in April 2013 (**Figures 6A,B**). As stratification started in May, bacterial communities from the epilimnion, metalimnion, and hypolimnion rapidly diverged and followed different successional trajectories. During late summer and fall, the bacterial community composition at 10 m depth developed towards the community composition of the sediment (**Figures 6A,C**). In particular, several bacterial genera with high relative abundances in the sediment throughout the year also became abundant at 10 m depth in late summer and fall (e.g., *Cytophaga, Clostridium, Smithella*; **Table 1**). During fall turnover in November and December, bacterial communities from 1 and 5 m became similar, but still differed from the bacterial community composition at 10 m depth (**Figure 6A**). Finally, in March 2014, the bacterial communities of all three water layers converged back to those in the spring of 2013. The NMDS further confirmed that, in contrast to the marked successional changes in the water column, the bacterial community in the sediment remained very stable during the seasons.

**Table 1:** Relative abundance (%) of bacterial genera in the sediment and in deeper parts of the water column (10 m depth), prior to the onset of water column stratification (March 6, 2013) and in the fall (November 7, 2013).

Genus	March, 2013		November, 2013	
	Sediment	10 m	Sediment	10 m
*Cytophaga*	10.93	0.02	10.76	1.26
*Syntrophus*	6.62	0.01	7.22	0.65
*Rhodocyclaceae*	3.69	0.70	5.49	0.28
*Methylobacter*	2.65	1.25	1.56	2.10
*Geobacter*	2.48	0.05	2.80	0.58
*Spirochaeta*	2.34	0.04	1.53	0.30
*Verrucomicrobium*	2.33	4.01	2.14	6.70
*Anaerophaga*	1.94	0.00	1.66	1.00
*Smithella*	1.50	0.00	1.11	2.27
*Desulfobacterium*	1.38	0.00	1.17	0.03
*Clostridium*	1.33	0.14	1.40	4.01
*Acidobacterium*	1.27	0.02	1.18	0.04
*Haliea*	1.19	0.01	1.51	0.00
*Methanosaeta*	1.08	0.01	0.56	0.00

*Only bacterial genera with a relative abundance >1% in the sediment are listed.*

### Environmental Variables Associated with Bacterial Succession

Redundancy analysis was applied to correlate the bacterial taxa with environmental variables. In total, 11 explanatory variables had a VIF < 10 including temperature, DO, PAR, pH, NH_4_^+^, NO_3_^-^, PO_4_^3-^, SO_4_^2-^, S^2-^, DOC and Cl^-^. Forward selection revealed that 6 of these 11 variables were significant in the redundancy analysis: DO, pH, NH_4_^+^, DOC, NO_3_^-^ and temperature (**Table 2**).

**Table 2:** Significance of the selected explanatory variables in the RDA correlation triplots (see **Figure 7**).

Explanatory Variable	AIC	Pseudo-*F*	*P*
DO	118.36	15.47	0.005
pH	121.73	11.32	0.005
NH_4_^+^	122.23	10.73	0.005
DOC	122.64	10.25	0.005
NO_3_^-^	127.42	4.92	0.005
Temperature	127.08	5.29	0.005

*The explanatory variables were selected by forward selection based on the pseudo-*F* statistic, using 9999 permutations to assess their significance. AIC = Akaike information criterion. Total variation explained by the RDA model was 48%.*

**FIGURE 7:**
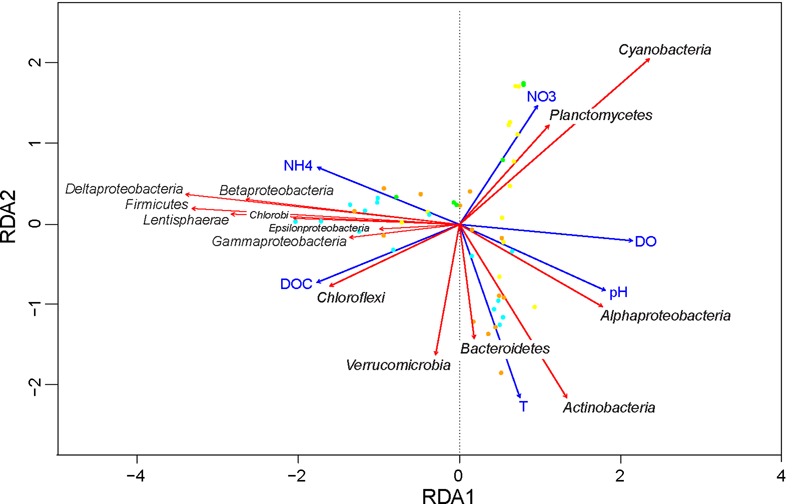
Redundancy analysis of the effect of environmental variables on bacterial community composition. Taxonomic responses (red arrows) are shown at the class level for *Proteobacteria* and at the phylum level for all other bacteria. Only taxa with a total average abundance above 0.1% are shown. All explanatory variables (blue arrows) in this triplot are significant (**Table 2**). Symbols represent the sampling points (yellow = spring; orange = summer; cyan = fall; green = winter). Total variation explained by the RDA model was 48%.

The first and second axis of the RDA plot explained 28.1% and 8.1% of the variation in the data, respectively (**Figure 7**). The first axis was positively correlated with DO and pH, but negatively correlated with NH_4_^+^, thus separating aerobic from anaerobic conditions. *Alphaproteobacteria* were associated with high DO and pH, whereas *Betaproteobacteria, Gammaproteobacteria, Deltaproteobacteria, Epsilonproteobacteria, Firmicutes, Chlorobi*, and *Lentisphaerae* were all associated with anaerobic conditions. Along the second axis, *Actinobacteria, Bacteroidetes*, and *Verrucomicrobia* were associated with the high temperatures in summer, whereas *Cyanobacteria* and *Planctomycetes* were associated with the high NO_3_^-^ concentrations in early spring. Finally, *Chloroflexi* were positively correlated with DOC concentration.

### Taxon–Taxon and Taxon–Environment Interactions

**FIGURE 8:**
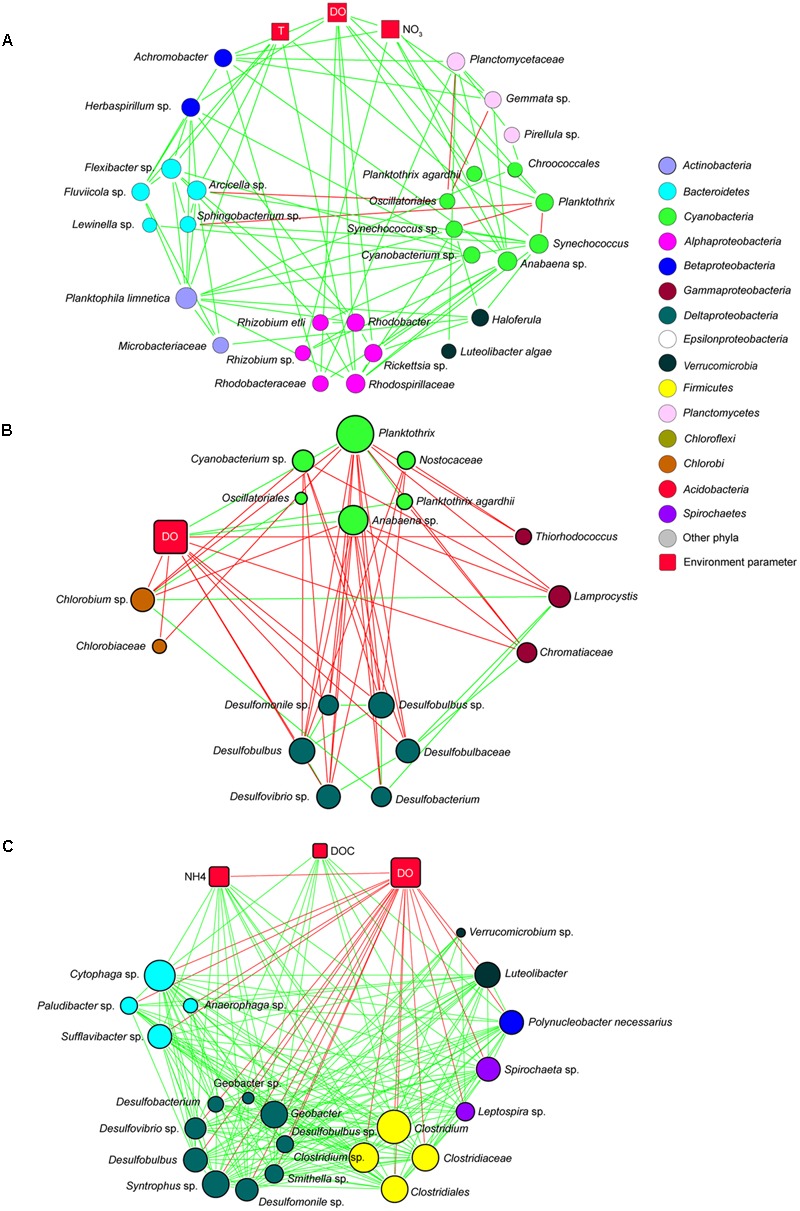
Co-occurrence subnetworks of bacterial species and environmental variables. **(A)** Subnetwork in the epilimnion; **(B)** subnetwork in the metalimnion; **(C)** subnetwork in the hypolimnion. Circles represent bacteria; red squares are environmental parameters. The size of the symbol indicates the number of interactions (‘degrees’). Green lines indicate positive interactions (co-occurrence); red lines indicate negative interactions (mutual exclusion).

Co-occurrence network analysis of samples from the water column resulted in a global network with 1931 taxon–taxon interactions and 335 taxon–environment interactions (Supplementary Figure S2). To visualize the results, subnetworks of the epilimnion, metalimnion, and hypolimnion were extracted from the global network. Bacterial species were assigned to a subnetwork only if they represented >0.1% of the OTUs in all samples of that subnetwork. Bacteria in the epilimnion were positively correlated with temperature, DO and NO_3_^-^ (**Figure 8A**). Most *Cyanobacteria* species had positive correlations with other bacteria in the epilimnion including species from *Actinobacteria, Alpha-* and *Betaproteobacteria, Bacteroidetes, Planctomycetes*, and *Verrucomicrobia*. In the metalimnion, *Cyanobacteria* were negatively correlated with *Chlorobi* (green sulfur bacteria), *Chromatiaceae* (purple sulfur bacteria), and *Deltaproteobacteria* and positively correlated with DO (**Figure 8B**). In contrast, *Chlorobi, Chromatiaceae*, and *Deltaproteobacteria* were positively correlated with each other, but negatively correlated with DO. In the hypolimnion, extensive positive correlations were observed between members of the *Firmicutes* (*Clostridiaceae*), *Deltaproteobacteria* (*Syntrophaceae, Desulfobulbaceae* and *Geobacteraceae*), *Bacteroidetes, Spirochaetes, Betaproteobacteria*, and *Verrucomicrobia* (**Figure 8C**). Most of these species were negatively correlated with DO and positively correlated with NH_4_^+^ and DOC.

## Discussion

### Mechanisms of Bacterial Succession

Our results point at a close coherence between seasonal stratification of the lake and seasonal changes in biogeochemical processes and microbial community structure.

In late winter and early spring, before the onset of stratification, nitrate concentrations were high throughout the water column while phosphate concentrations were low. The ratio of dissolved inorganic nitrogen to phosphorus (DIN:DIP >75) greatly exceeded the canonical Redfield ratio of 16:1, which indicates that P was the major limiting factor for the phytoplankton spring bloom. During this period, the bacterial community was dominated by *Cyanobacteria* and aerobic heterotrophic bacteria of the phylum *Planctomycetes*, which were both dispersed throughout the water column. The cyanobacterial community consisted largely of the non-nitrogen-fixing *Planktothrix agardhii*, a filamentous species of eutrophic lakes that often dominates in turbid and well-mixed waters with high N:P ratios (Mur et al., 1999; Dokulil and Teubner, 2000).

During summer stratification, nitrate and phosphate concentrations were both depleted in the epilimnion, indicating that phytoplankton growth was co-limited by both nutrients during summer. *Cyanobacteria* had a much lower relative abundance than in spring, and in the epilimnion they were replaced by *Alphaproteobacteria, Bacteroidetes*, and *Actinobacteria*. Stratification of the lake created anoxic conditions in the hypolimnion, most likely caused by microbial degradation of organic matter, which resulted in higher DOC and NH_4_^+^ concentrations and lower pH during the stratification period. A wide variety of anaerobic heterotrophic bacteria, including members of the *Bacteroidetes, Betaproteobacteria, Deltaproteobacteria, Firmicutes*, and *Spirochaetes*, were abundant in the anoxic hypolimnion. The decrease in sulfate and concomitant increase in sulfide concentration is consistent with the activity of sulfate-reducing *Deltaproteobacteria* in the hypolimnion (e.g., *Desulfobulbaceae, Syntrophaceae*; **Figure 8C**). The upward diffusion of sulfide and downward flux of light provided suitable growth conditions for purple sulfur bacteria (*Chromatiaceae*, dominant in the *Gammaproteobacteria*) in the metalimnion and green sulfur bacteria (*Chlorobi*) in both the meta- and hypolimnion.

Mixing of the lake led to hypoxia throughout the entire water column during fall turnover. Sulfide rapidly vanished from the deeper water layers during the fall, indicative of the activity of sulfur-oxidizing bacteria (e.g., members of the *Epsilonbacteria*; Campbell et al., 2006). *Chlorobi, Chromatiaceae* (*Gammaproteobacteria*), *Deltaproteobacteria*, and *Firmicutes* all disappeared during fall turnover, whereas *Polynucleobacter* (*Betaproteobacteria*) and *Methylobacter* (*Gammaproteobacteria*) spread out from the former meta- and hypolimnion into the surface layer of the lake. These observations are quite similar to recent findings of Pjevac et al. (2015), who studied a stratified seawater lake that also became completely anoxic after mixing of the water column. Similar to our study, they also observed that phototrophic sulfur bacteria (*Chlorobi* and *Chromatiaceae*) disappeared after holomixis. In their study, however, the anoxic water column remained sulfidic and became dominated by gammaproteobacterial sulfur oxidizers. In our study, the lake became nonsulfidic during fall turnover, which led to a community dominated by *Polynucleobacter* and gammaproteobacterial methane oxidizers (*Methylobacter*). Ammonium that had accumulated in the hypolimnion during summer stratification was oxidized to nitrate during winter mixing, which fueled a new bloom of non-nitrogen-fixing *Cyanobacteria* (*Planktothrix* spp.) in the next early spring.

Overall, these synchronous trends indicate that the seasonal succession of bacterial communities is closely associated with seasonal changes in environmental variables and quite predictable, providing a Clementsian view on microbial succession in stratified lakes.

### Divergence and Convergence of Bacterial Communities

In line with expectation, the bacterial community composition in epilimnion, metalimnion and hypolimnion diverged during summer stratification. In the epilimnion, *Actinobacteria* became dominant. In the metalimnion, a community of *Betaproteobacteria, Gammaproteobacteria* (mainly purple sulfur bacteria of the *Chromatiaceae*), and *Deltaproteobacteria* developed. The hypolimnion was dominated by members of the *Betaproteobacteria, Deltaproteobacteria, Firmicutes*, and *Chlorobi* (green sulfur bacteria). A similar divergence of bacterial communities in the epilimnion and hypolimnion has been reported for several other seasonally stratified lakes (Salcher et al., 2008; Shade et al., 2008; Garcia et al., 2013; Paganin et al., 2013; Yu et al., 2014; Okazaki and Nakano, 2016; Schmidt et al., 2016).

Changes in bacterial community composition during fall turnover have received far less attention, presumably because one would expect a straightforward homogenization of the bacterial communities. Yet, two aspects are quite noteworthy in this lake. First, the community composition that emerged upon fall turnover did not mimic the average of the microbial communities that dominated the different water layers in the preceding weeks. Instead, many of the bacterial taxa that were abundant during summer stratification disappeared (e.g., *Actinobacteria, Chlorobi, Chromatiaceae, Deltaproteobacteria, Firmicutes*), whereas other taxa already present in the hypolimnion became dominant during fall turnover (e.g., *Polynucleobacter, Methylobacter*). Hence, mixing of the lake led to major shifts in community composition.

Second, the bacterial community of Lake Vechten did not fully homogenize across the entire depth of the water column during fall turnover. Instead, *Polynucleobacter* and *Methylobacter* spread out to the upper half of the water column, whereas *Epsilonproteobacteria* became dominant in remnants of the hypolimnion. This spatial variation in bacterial community composition persisted in November and December despite a nearly uniform temperature profile. Vertical mixing in the surface layers of lakes is often relatively fast due to wind action and convective cooling (Imberger, 1985; Tedford et al., 2014), but vertical mixing toward deeper water layers of lakes can be a slow and incomplete process (MacIntyre, 1993; Imboden and Wüest, 1995). The depth profiles of pH, ammonium and most other physico-chemical parameters show that, in December, the surface mixed layer spanned the upper 7–8 m of the water column. The pH remained lower and ammonium concentration was higher below 8 m depth, indicating that mixing of the lake during the fall was not complete, which provided a distinct niche for *Epsilonproteobacteria* in the deeper water layer. *Epsilonproteobacteria* often play a key role in the oxidation of sulfur and other reduced compounds at low oxygen levels, and can occur in high abundances at oxic-anoxic interfaces (Campbell et al., 2006). Only in early spring did mixing of oxygen-rich water reach to the deeper parts of the lake and the bacterial community converged to a similar species composition across almost the entire depth gradient.

### Bacteria in the Sediment

The bacterial composition in the sediment of Lake Vechten showed much less seasonal variation than that in the water column. Yet, the sediment community was quite diverse, consisting of *Beta*-, *Gamma-* and *Deltaproteobacteria, Bacteroidetes, Firmicutes, Verrucomicrobia, Spirochaetes*, and a variety of other taxa. Dominant bacterial phyla and classes in the sediment of Lake Vechten, such as *Beta*-, *Gamma-* and *Deltaproteobacteria, Bacteroidetes*, and *Firmicutes*, have also been found in other freshwater lake sediments (e.g., Ding et al., 2015; Zhang et al., 2015; Dai et al., 2016). *Verrucomicrobia* were relatively abundant in the sediment of Lake Vechten whereas they had not been detected in Dianchi Lake and Erhai Lake (Dai et al., 2016). In contrast, *Actinobacteria* only constituted 1% of the bacterial community and *Chlorobi* were almost negligible in the sediment of Lake Vechten, whereas they can be quite abundant in the sediments of other freshwater lakes (Zhang et al., 2015; Dai et al., 2016; Zeng et al., 2016). Hence, the sediments of freshwater lakes have several bacterial groups in common, yet the bacterial community composition still varies considerably among lakes, possibly in association with differences in trophic status, redox conditions, and a variety of other sediment characteristics (Ding et al., 2015; Dai et al., 2016; Zeng et al., 2016).

While the bacterial community composition in the hypolimnion diverged from the epilimnion community during the summer stratification, it became more similar to the community composition in the sediment, especially in the fall. For instance, proportions of *Firmicutes* and *Verrucomicrobia* were similar in the hypolimnion and the sediment. Yet, there were still differences between hypolimnion and sediment, as *Betaproteobacteria* and *Gammaproteobacteria* were dominant bacterial groups in the hypolimnion, whereas *Bacteroidetes* and *Deltaproteobacteria* were the most abundant bacterial groups in the sediment. Convergence of bacterial communities in the hypolimnion to those in the sediment indicates that the sediment might serve as a ‘seed bank’ (*sensu* Lennon and Jones, 2011) of anaerobic bacteria, such as *Clostridium* (*Firmicutes*) and *Smithella* (*Deltaproteobacteria*) (**Table 1**). These anaerobic bacteria were confined to the sediment when the water column was oxic in spring, increased in the anoxic hypolimnion during summer and fall, and again withdrew to the sediment once the complete water column became oxic in winter.

### Development of Anoxia

Stratification of the lake led to anoxic conditions in the hypolimnion during the summer period, similar to previous observations of Lake Vechten in the 1970s and 1980s (Blaauboer, 1982; Steenbergen, 1982; Steenbergen and Verdouw, 1982; Verdouw and Dekkers, 1982).

However, we are not aware of earlier reports of anoxia spreading to the surface layer of Lake Vechten during fall turnover. In the 1970s and 1980s, when the lake was extensively monitored, the hypolimnion was usually anoxic and sulfidic from late May till late October, and was subsequently oxygenated during fall turnover in mid November (Steenbergen and Verdouw, 1982; Sweerts et al., 1991). We observed an anoxic and sulfidic hypolimnion that lasted slightly longer, from early May till early November 2013. Subsequently, the entire water column became anoxic when the lake was mixed during fall turnover in early December (**Figure 2B**), although sulfide largely vanished from the water column (**Figure 2H**).

Increases in the extent and duration of anoxia in aquatic ecosystems are commonly attributed to eutrophication and enhanced stratification by global warming (Diaz and Rosenberg, 2008; Meire et al., 2013; Jenny et al., 2016). Winter concentrations of dissolved nitrogen and phosphorus measured during our 2013–2014 study were quite similar to the winter concentrations measured in the 1970s (Steenbergen and Verdouw, 1982). Hence, there is no evidence for recent eutrophication of the lake. The second half of October 2013 was exceptionally warm, however, with maximum air temperatures above 20°C during daytime and above 12°C at night (which is 6°C higher than the 30-year averages of both daytime and nighttime temperature). These high autumn temperatures provide a plausible explanation for the prolonged stratification period, well into November of 2013, which may have led to a further lowering of the redox potential in the hypolimnion and to subsequent anoxia during fall turnover (cf. Nürnberg, 1995). These results may offer a glimpse of future lake responses to global warming, as an earlier onset and longer duration of the summer stratification is consistent with predictions of climate models (Stefan et al., 1998; Peeters et al., 2002) and observations of other European lakes (Livingstone, 2003; Thackeray et al., 2008; Arvola et al., 2010).

## Author Contributions

JH and GM designed the study. MD, RS, KK, and GM performed the sampling and data analysis. MD, JH, and GM wrote the manuscript.

## Conflict of Interest Statement

The authors declare that the research was conducted in the absence of any commercial or financial relationships that could be construed as a potential conflict of interest.
