# Supplementary material for: Succession of Bacterial Communities in a Seasonally Stratified Lake with an Anoxic and Sulfidic Hypolimnion
Source: Front Microbiol. 2017 Dec 14;8:2511. doi: 10.3389/fmicb.2017.02511 (PMC5735980; doi:10.3389/fmicb.2017.02511)
Supplement: Supplementary file 1 [file Data_Sheet_1.docx]

Supplementary Material

**Succession of bacterial communities in a seasonally stratified lake**

**with an anoxic and sulfidic hypolimnion**

Muhe Diao, Ruben Sinnige, Karsten Kalbitz, Jef Huisman, Gerard Muyzer^*^

* Correspondence: g.muijzer@uva.nl

This file includes:

**Supplementary Figures**

**Figure S1.** Composition of the bacterial community over time and space in Lake Vechten.

**Figure S2.** Global co-occurrence network of bacterial species and environmental variables.

**Supplementary Tables:**

**Table S1.** List of DNA samples used for 16S rRNA gene amplicon sequencing.

**Table S2.** Relative abundances of major bacterial taxa in the water column and sediment.


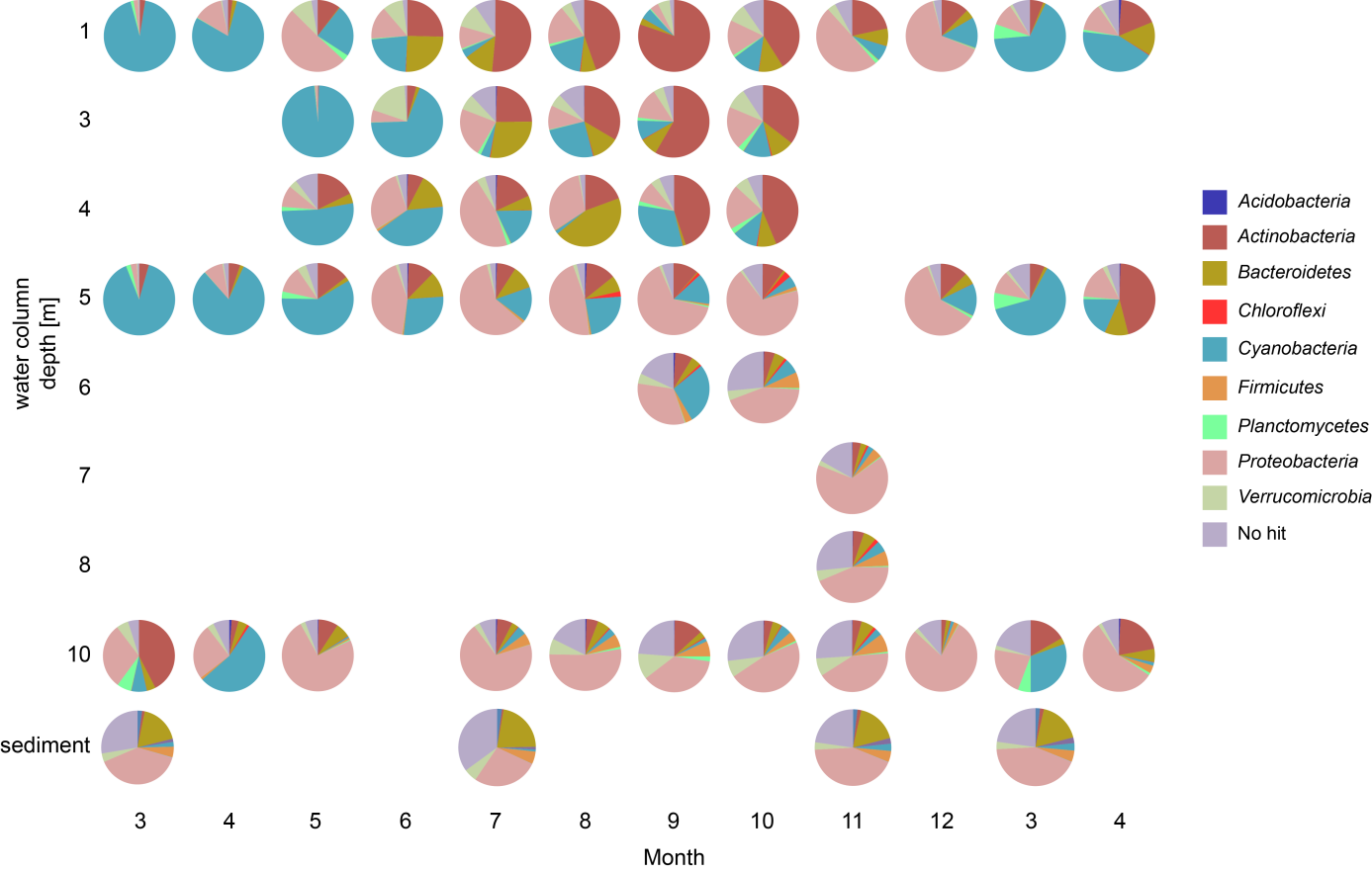


**Supplementary Figure S1.** Composition of the bacterial community over time and space in Lake Vechten. The lower row presents the bacterial community composition in the sediment. Only bacterial phyla with an average relative abundance (sum of all relative abundances divided by sample number) > 0.3% are shown.


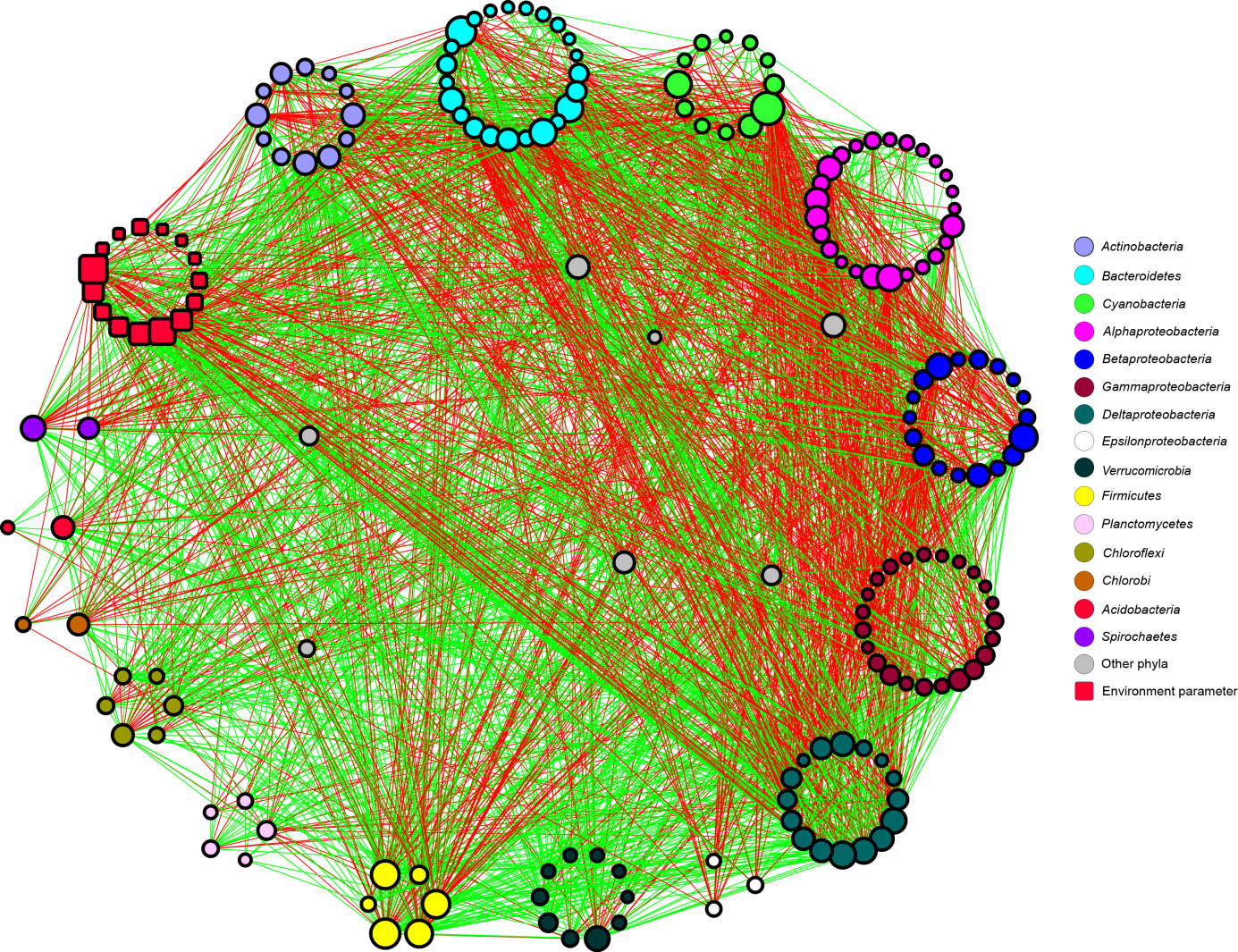


**Supplementary Figure S2.** Global co-occurrence network of bacterial species and environmental variables. Circles represent bacteria; red squares are environmental parameters. The size of the symbol indicates the number of interactions ('degrees'). Green lines indicate positive interactions (co-occurrence); red lines indicate negative interactions (mutual exclusion).

**Supplementary Tables**

**Supplementary Table S1**. List of DNA samples used for 16S rRNA gene amplicon sequencing.

| **Sample ID** | **Date** | **Depth (m)** |  | **Sample ID** | **Date** | **Depth (m)** |
| --- | --- | --- | --- | --- | --- | --- |
|  |  |  |  |  |  |  |
| A0301 | 2013-03-06 | 1 |  | G0901 | 2013-09-02 | 1 |
| A0305 |  | 5 |  | G0903 |  | 3 |
| A0310 |  | 10 |  | G0904 |  | 4 |
| A03S |  | Sediment |  | G0905 |  | 5 |
| B0401 | 2013-04-10 | 1 |  | G0906 |  | 6 |
| B0405 |  | 5 |  | G0910 |  | 10 |
| B0410 |  | 10 |  | H0901 | 2013-09-30 | 1 |
| C0501 | 2013-05-08 | 1 |  | H0903 |  | 3 |
| C0503 |  | 3 |  | H0904 |  | 4 |
| C0504 |  | 4 |  | H0905 |  | 5 |
| C0505 |  | 5 |  | H0906 |  | 6 |
| C0510 |  | 10 |  | H0910 |  | 10 |
| D0601 | 2013-06-06 | 1 |  | I1101 | 2013-11-07 | 1 |
| D0603 |  | 3 |  | I1107 |  | 7 |
| D0604 |  | 4 |  | I1108 |  | 8 |
| D0605 |  | 5 |  | I1110 |  | 10 |
| D0610 |  | 10 |  | I11S |  | Sediment |
| E0701 | 2013-07-01 | 1 |  | J1201 | 2013-12-03 | 1 |
| E0703 |  | 3 |  | J1205 |  | 5 |
| E0704 |  | 4 |  | J1210 |  | 10 |
| E0705 |  | 5 |  | K0201 | 2014-02-25 | 1 |
| E0710 |  | 10 |  | K0205 |  | 5 |
| E07S |  | Sediment |  | K0210 |  | 10 |
| F0701 | 2013-07-29 | 1 |  | K02S |  | Sediment |
| F0703 |  | 3 |  | L0301 | 2014-03-31 | 1 |
| F0704 |  | 4 |  | L0305 |  | 5 |
| F0705 |  | 5 |  | L0310 |  | 10 |
| F0710 |  | 10 |  |  |  |  |

**Supplementary Table S2.** Relative abundances of major bacterial taxa in the water column and sediment.

| Bacterial taxa | Water column | | |  | Sediment | | |
| --- | --- | --- | --- | --- | --- | --- | --- |
|  |  | | |  |  | | |
|  | Average (%) | Max. (%) | Min. (%) |  | Average (%) | Max. (%) | Min. (%) |
| *Actinobacteria* | 18.2 | 79.9 | 0.4 |  | 1.0 | 1.1 | 0.5 |
| *Bacteroidetes* | 6.8 | 44.6 | 0 |  | 17.5 | 20.3 | 16.4 |
| *Cyanobacteria* | 26.9 | 98.0 | 0.8 |  | 1.9 | 2.9 | 0.8 |
| *Alphaproteobacteria* | 4.4 | 38.4 | 0.3 |  | 1.3 | 1.7 | 0.8 |
| *Betaproteobacteria* | 18.2 | 62.7 | 0.1 |  | 8.5 | 11.6 | 4.6 |
| *Gammaproteobacteria* | 6.0 | 28.9 | 0.2 |  | 5.9 | 7.4 | 3.4 |
| *Deltaproteobacteria* | 0.9 | 5.1 | 0 |  | 17.0 | 18.4 | 14.5 |
| *Epsilonproteobacteria* | 1.9 | 57.3 | 0 |  | 0 | 0 | 0 |
| *Verrucomicrobia* | 4.0 | 18.6 | 0.1 |  | 3.7 | 5.0 | 2.9 |
| *Firmicutes* | 1.3 | 7.9 | 0 |  | 4.4 | 4.8 | 4.2 |
| *Planctomycetes* | 1.4 | 7.4 | 0.1 |  | 0.2 | 0.2 | 0.1 |
| *Chloroflexi* | 0.4 | 2.9 | 0 |  | 1.4 | 2.0 | 1.0 |
| *Chlorobi* | 0.1 | 1.1 | 0 |  | 0.2 | 0.2 | 0.2 |
| *Lentisphaerae* | 0.2 | 2.0 | 0 |  | 0.3 | 0.4 | 0.2 |
| *Spirochaetes* | 0.1 | 0.6 | 0 |  | 2.7 | 3.5 | 2.0 |
| *Fusobacteria* | 0.1 | 0.8 | 0 |  | 0 | 0 | 0 |

The table shows the average over all samples, and the minimum and maximum relative abundance observed.
